# Links between plant and fungal diversity in habitat fragments of coastal shrubland

**DOI:** 10.1371/journal.pone.0184991

**Published:** 2017-09-19

**Authors:** Mia R. Maltz, Kathleen K. Treseder, Krista L. McGuire

**Affiliations:** 1 Center for Conservation Biology, University of California Riverside, Riverside, California, United States of America; 2 Department of Ecology and Evolutionary Biology, University of California Irvine, Irvine, California, United States of America; 3 Department of Biology, University of Oregon, Eugene, Oregon, United States of America; Chinese Academy of Forestry, CHINA

## Abstract

Habitat fragmentation is widespread across ecosystems, detrimentally affecting biodiversity. Although most habitat fragmentation studies have been conducted on macroscopic organisms, microbial communities and fungal processes may also be threatened by fragmentation. This study investigated whether fragmentation, and the effects of fragmentation on plants, altered fungal diversity and function within a fragmented shrubland in southern California. Using fluorimetric techniques, we assayed enzymes from plant litter collected from fragments of varying sizes to investigate enzymatic responses to fragmentation. To isolate the effects of plant richness from those of fragment size on fungi, we deployed litter bags containing different levels of plant litter diversity into the largest fragment and incubated in the field for one year. Following field incubation, we determined litter mass loss and conducted molecular analyses of fungal communities. We found that leaf-litter enzyme activity declined in smaller habitat fragments with less diverse vegetation. Moreover, we detected greater litter mass loss in litter bags containing more diverse plant litter. Additionally, bags with greater plant litter diversity harbored greater numbers of fungal taxa. These findings suggest that both plant litter resources and fungal function may be affected by habitat fragmentation’s constraints on plants, possibly because plant species differ chemically, and may thus decompose at different rates. Diverse plant assemblages may produce a greater variety of litter resources and provide more ecological niche space, which may support greater numbers of fungal taxa. Thus, reduced plant diversity may constrain both fungal taxa richness and decomposition in fragmented coastal shrublands. Altogether, our findings provide evidence that even fungi may be affected by human-driven habitat fragmentation via direct effects of fragmentation on plants. Our findings underscore the importance of restoring diverse vegetation communities within larger coastal sage scrub fragments and suggest that this may be an effective way to improve the functional capacity of degraded sites.

## Introduction

Microbial processes regulate decomposition rates and the release of soil C pools into the atmosphere in the form of CO_2_ [1]. However, microbial processes are threatened by land-use changes [2], which result in habitat fragmentation and biodiversity loss [3–6]. By physically separating biological communities into habitats of different sizes, fragmentation may exacerbate this biodiversity loss and alter ecosystem function [7, 8]. Although previous studies have shown that human-driven habitat fragmentation reduces biodiversity or ecosystem function, most of these studies have been conducted on macroscopic organisms such as birds [9] or vascular plants [10] without sufficient emphasis on microbes [11–13]. Fungi in particular are considered the engines of nutrient recycling and thus support numerous ecosystem functions [14, 15]. Given their functional role in the ecosystem [16–18], it is important to understand more precisely how fragmentation affects fungi.

Determining how fungi respond to habitat fragmentation may be critical for managing fragmented ecosystems, yet our understanding of how habitat fragmentation may affect fungal communities or hinder fungal processes is relatively limited [19–21]. Evidence suggests that fragmentation lowers root-associated fungal diversity and abundance, possibly because reductions in host-plant diversity or performance may constrain plant-associated fungal communities or limit resources provided to fungi by their host-plants. In central Argentina, Grilli and colleagues [20] examined how fragmentation affects root-associated fungal communities and found more dark-septate endophytic and arbuscular mycorrhizal fungi in larger forest fragments than was found in smaller fragments. Other studies in northern California forests examining the effect of fragmentation on root-associated fungi reported strong species-area relationships among ectomycorrhizal fungi within patchy host-tree matrices [22, 23]. Previous studies have also examined fragmentation effects on microbial communities using experimental microcosms or experimentally fragmented ecosystems. These studies indicate that fragmentation can indirectly affect fungal growth rates [5, 24] or N cycling [5, 25]. Although it is challenging for these experiments to fully replicate the complexity of fragmented ecosystems, these studies highlight how habitat fragmentation may affect fungal function.

Fragmentation often limits the number of plant species (i.e., plant species richness) in small fragments [10, 26, 27] by reducing ecological niche space [28]. Fragmentation may also increase within fragment heterogeneity, which often reduces suitable habitat area [29], or alters habitat complexity [30]. Smaller fragments may harbor smaller populations containing fewer individuals than their minimum viable population size [31], leading to increased population vulnerability or species loss [32]. Reductions in plant diversity may in turn affect plant litter abundance and composition, as well as decomposer fungal production of enzymes for decaying plant litter. Additionally, enzyme production and fungal processes may influence how quickly woody debris decomposes or accumulates, which could have implications for both soil aggregation [33] and wildfire regimes [34] in these ecosystems.

Fungi and the extracellular enzymes they produce serve important ecological functions; these enzymes are considered to be rate-controlling agents of decomposition [35]. Decomposition of plant litter is driven by a successional loop in which fungal communities and extracellular enzymes are linked to plant litter substrates [36, 37]. In this loop, the composition of plant litter substrates may select for fungal taxa that produce extracellular enzymes, which modify a particular substrate. The downstream products and byproducts of degradation may likewise select for different taxa capable of modifying other substrates within decaying litter. Because plant species differ in their litter chemistries and decompose at different rates, diverse plant litter mixtures are likely to contain a variety of substrates at different stages of decay at any given time. This increased variety of substrates [38] and the complex interactions among both enzymes and substrates [39] may not only enhance potential extracellular enzyme activity, but may also increase microhabitat heterogeneity and diversify fungal growth forms [40].

In fragmented habitats, with fewer numbers of plant species, there may be only a narrow range of litter substrates for fungi, which may in turn reduce fungal activity or inhibit fungal processes. Indeed, the subset of plant species within smaller fragments may differ physically or chemically from plant assemblages in larger fragments, which could alter litter quality and decomposition. Thus, human-driven habitat fragmentation may not only alter litter composition by reducing plant diversity [26] but may also affect extracellular enzyme activity by changing the functional capacity of fungal communities [41].

In small habitat fragments with low plant diversity, the relative influence of plant species richness on fungi and plant litter decay may be difficult to isolate from the effects of habitat fragment size on fungi [38]. In fact, the consequences of reduced plant diversity in a small fragment may be as important as the size of that fragment in determining fungal function. Elucidating the mechanisms driving fungal function may be crucial for managing habitat remnants within fragmented ecosystems.

Southern California’s coastal sage scrub ecosystems are prime examples of fragmentation [42, 43]. These coastal California shrublands provide habitat for wildlife, and are characterized by high degrees of endemism and species richness. Therefore, evaluating the impacts of habitat fragmentation and reduced plant diversity on biotic communities in southern California’s threatened coastal sage scrub ecosystems would be especially valuable. These coastal sage scrub ecosystems are becoming increasingly rare due to urban and agricultural development and anthropogenic N deposition [44, 45]. In fact, only about 10% of coastal sage scrub ecosystems are still intact [46], making these plant communities arguably the most endangered habitat in the United States [47]. Fragmentation affects coastal sage scrub-associated biotic communities [42], but the linkages between coastal sage scrub plant diversity and fungal function remain poorly understood. Therefore, understanding fungal dynamics and plant litter decay in coastal sage scrub habitats may be especially important for managing these fragmented ecosystems.

In order to examine the effects of habitat fragmentation on plant species richness and fungal function, and to evaluate the extent to which fungi are influenced by plant species richness in fragmented ecosystems, we addressed the following questions: (1) Does habitat fragmentation reduce plant diversity and fungal function? (2) Does plant species richness control fungal taxa richness and fungal function? To answer these questions, we investigated the effects of habitat fragment size on plant richness and fungal function in fragmented coastal sage scrub ecosystems. In order to evaluate fungal function, we quantified the potential activity of three extracellular enzymes important in either chitin or cellulose degradation within plant litter from habitat fragments of varying sizes. Next, we manipulated plant litter richness in order to isolate the effect of reduced plant species richness on fungal taxa richness and decomposition. We hypothesized that (1) plant species richness and fungal function decline with habitat fragment size and that (2) plant litter diversity controls fungal taxa richness and fungal function in fragmented coastal sage scrub habitats. Specifically, if plant litter diversity controls fungal taxa richness and fungal function then plant litter composed of greater numbers of plant species would likely decay more rapidly and harbor more fungal taxa than litter from fewer plant species.

## Materials and methods

### Site descriptions

**Fig 1:**
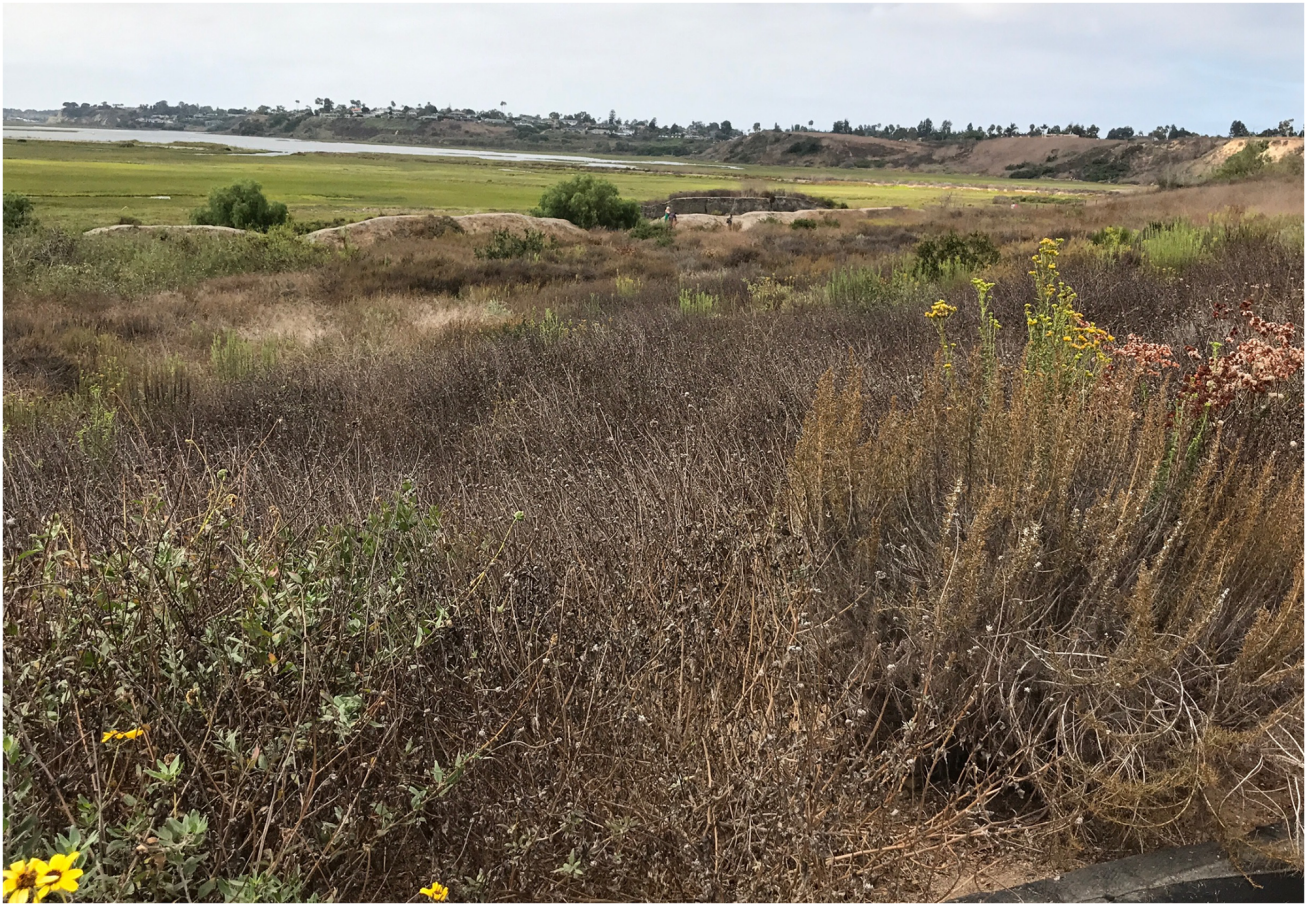
Coastal sage shrubland. Coastal sage scrub habitat and native vegetation in southern California.

We investigated the effects of habitat fragmentation and plant richness on fungal function in a native, fragmented, coastal sage scrub habitat (Fig 1) at Newport Back Bay in southern California (33° 37’ 35” N, 117° 53’ 30” W). We were granted permits from both the California Department of Fish and Wildlife and the California Coastal Commission to conduct our study at Newport Back Bay. Soils at the sites are classified as Typic Palexeralfs and Typic Xerorthents belonging to the Myford and Cieneba series [48, 49]. Soils are moderately to excessively well-drained sandy loams with neutral to slightly acidic pH (~ pH 6.0—pH 6.8).

Southern California coastal sage scrub is a drought-deciduous vegetation community composed of aromatic shrubs. The canopy of these relatively low-growing shrubs extends to less than one m in height. Numerous taxa depend on sage scrub habitat. In fact, coastal sage scrub canopies provide the sole habitat for the nesting songbird *Polioptila californica* (Muscicapidae), which has been listed as threatened under the United States Endangered Species Act [46].

The dominant shrub species in our coastal sage scrub fragments are *Artemisia californica* (Asteraceae Less.), *Eriogonum fasciculatum* (Polygonaceae Benth.), *Encelia californica* (Asteraceae Nutt.), *Cleome isomeris* (Cleomaceae Greene), *Rhus integrifolia* (Anacardiaceae Nutt. Brewer & Watson), *Isocoma menziesii* (Asteraceae Hook. & Arn. Nesom), and *Salvia apiana* (Lamiaceae Jeps.). Non-native plants are present in areas adjacent to and at the edges of our fragments, including *Brassica nigra* (Brassicaceae L. Koch), *Salsola kali* (Chenopodiaceae L.), *Cynara cardunculus* (Asteraceae L.), and *Schinus terebinthifolius* (Anacardiaceae Raddi). The largest habitat fragment contained: *A*. *californica*, *B*. *nigra*, *C*. *cardunculus*, *I*. *menziesii*, *R*. *integrifolia*, *S*. *apiana*, and *S*. *terebinthifolius*. The smaller fragments contained most of these same plant species, however, *C*. *isomeris* and *E*. *californica* were just found in Fragment 2, and *Eriogonum fasciculatum* was only in Fragments 4, 5, and 6 (Table 1).

**Table 1:** Native plant species in our fragments.

Native plant species	Common name	Botanical family	Found in fragment
*Artemisia californica*	California coastal sagebrush	Asteraceae	1, 2, 3, 4
*Eriogonum fasciculatum*	California buckwheat	Polygonaceae	4, 5, 6
*Encelia californica*	California brittlebrush	Asteraceae	2
*Cleome isomeris*	California bladderpod	Cleomaceae	2
*Rhus integrifolia*	lemonade sumac	Anacardiaceae	1, 2
*Isocoma menziesii*	Menzies’ goldenbush	Asteraceae	1, 3
*Salvia apiana*	white sage	Lamiaceae	1

Surrounding these fragments are open grounds and walking trails. This site experiences a Mediterranean climate with warm dry summers and cool wet winters. The mean annual temperature is 16.6°C. Mean annual precipitation is 295 mm with the majority of precipitation occurring between November and March (http://www.nws.noaa.gov/).

### Fragment size

To investigate the effects of habitat fragmentation on fungal function, we established plots within a series of six coastal sage scrub fragments in the Newport Back Bay. These fragments were composed of one relatively large fragment (Fragment 1: 5415 m^2^) and five smaller fragments (Fragment 2: 1234 m^2^, Fragment 3: 684 m^2^, Fragment 4: 186 m^2^, Fragment 5: 26 m^2^, and Fragment 6: 23 m^2^).

Plant species richness in each fragment was measured by vegetative monitoring and recording each species present in each fragment. We refrained from sampling 1 m from fragment edges to account for edge effects. Via direct counts and exhaustive vegetation sampling, we determined that plant species richness increased with fragment size (R^2^ = 0.976; p < 0.001).

We created five 5 m transects from the southwest corner of each fragment and used a random number generator to select coordinates along the transect. Then, we collected ten surface litter samples at these coordinates, where litter was present. Each collection contained litter from at least one plant species, however many collections contained dry intact litter from aggregated litter mixtures. Samples were immediately frozen on dry ice for transport back to the laboratory at University of California, Irvine. Samples were thawed and composited in the laboratory, in order to combine technical replicates into representational samples. We assessed extracellular enzyme activity in each composited litter sample by conducting assays on 0.1 g litter samples from each fragment.

From each litter sample, we assessed potential extracellular enzyme activity for the chitinase enzyme *N*-acetyl-glucosaminidase and two cellulase enzymes, β-glucosidase and cellobiohydrolase, involved in plant litter degradation [50] (Table 2). We added 0.1 g of litter to 60 ml of 25 mM maleate buffer solution (pH 6.0). To create homogenate solutions, we homogenized our litter samples using a Biospec Tissue Tearer (14 mm generator) for four 30 second pulses with 30 second intervals between each pulse. Then we pipetted 200 ml of stirred homogenate per well into eight replicate wells within a 96-well microplate. We added 50 μl of 1000 μM substrate solutions to each homogenate well; each substrate solution was prepared from 4-methylumbelliferone (MUB) fluorescent dye-conjugated substrates specific to each enzyme (Table 2). We corrected for background fluorescence with homogenate control wells (without substrate solutions added) and substrate control wells (without added homogenate samples). Black micro-plates were covered for one hour; after incubation, we added 10 μl of 1.0 NaOH to stop reactions.

**Table 2:** Extracellular enzymes assayed from litter.

Enzyme	Function	Substrate
**β-glucosidase**	Cellulose degradation	4-MUB-β-D-glucopyraniside
**Cellobiohydrolase**	Cellulose degradation	4-MUB-β-D-cellobioside
**N-acetyl-β-D-glucosaminidase**	Chitin degradation	4-MUB-N-acetyl-β-D-glucosaminide

Extracellular enzymes assayed from litter from a fragmented coastal sage scrub ecosystem; functions and corresponding substrates for these enzymes.

We conducted fluorimetric assays (as detailed in [51], protocols.io dx.doi.org/10.17504/protocols.io.jg3cjyn) for each of three hydrolytic enzymes: β-glucosidase and cellobiohydrolase, and *N*-acetyl-glucosaminidase. We measured fluorescence at 365 nm excitation and 450 nm emission. From each sample, we recorded fluorescence values for MUB substrate (substrate control), homogenate (homogenate control), MUB standards in the presence of maleate buffer (standard), and MUB in the presence of homogenate. We calculated potential extracellular enzyme activity as per [50] as:

$$Activity\;\left(nmol\;g^{-1}h^{-1}\right)=\frac{\text{Net fluorescence }\times \text{ Buffer volume }\left(\text{ml}\right)}{\text{Emission coefficient }\times \text{ Homogenate volume }\left(\text{ml}\right)\;\times \text{ Time }\left(\text{hours}\right)\;\times \text{ Litter mass }\left(\text{g}\right)}$$

### Decomposition experiment

Our plant litter manipulation experiment allowed us to isolate the effects of plant diversity on fungal taxa richness and function from the effects of fragment size on these response variables. We experimentally manipulated plant litter diversity on the soil surface in our largest coastal sage scrub fragment using a mixture of plant species that were representative of those found in the fragments (Table 1). For this experiment, we constructed 20 cm x 20 cm mesh bags from 1 mm nylon mesh (i.e., litter bags; Millipore, Bradford MA, USA) and added 2 g of air-dried leaf litter collected from our fragments into each bag. Prior to assembling our litter bags, we sterilized the leaf litter via gamma irradiation [52] (UC Irvine Medical Center, Irvine CA) at 2.5–3.0 Mrad for 48 hours.

Using a replacement design [53], we randomly assembled the leaf litter into mixtures composed of either one single plant species, three mixed species, five mixed species, or seven mixed species. Each single-species litter bag contained only one of each of the seven plant species. Given that decomposition rates of the individual species may have differed, our mixed-species litter bags contained randomized mixtures of plant litter comprised of plant species found in our fragments. The numbers of plant species in each of our mixed-species litter bags were representative of a plant diversity level found in one of our habitat fragments; each mixed-species litter bag contained equally divided plant litter, as per a replacement series.

We cleared all standing litter from a 2 m^2^ randomly-located plot within the largest habitat and deployed these litter bags in a randomized block design with seven replicates per plant litter diversity level, resulting in a total of 28 litter bags within the plot. These nylon litter bags allowed nutrients, microbes, fungi, and micro-fauna to move freely into and out of the litter bags.

After one year of field incubation, we harvested these litter bags and weighed each litter bag to determine the percent leaf litter mass remaining in each bag. Although settled dust or soil may have added mass to litter bags, any consequence of this settlement would have similarly influenced all treatments within individual blocks. As a measure of decomposition, we calculated percent litter mass remaining (%) as the mass of oven-dried litter (g) in each bag after one year of field incubation divided by the initial mass of dried litter (g) in that litter bag.

Litter mass was then subsampled to assay for fungal taxa richness as per [54]. We weighed 0.25 g aliquots of each litter sample in triplicate. We extracted total DNA from each of the three 0.25 g litter aliquots using a Powersoil DNA extraction kit (MoBio Carlsbad, CA) and pooled these DNA extracts into a single representative DNA extract. DNA concentrations were standardized to 10 ng/μl before PCR amplification. We targeted a portion of the 18S rRNA gene using universal fungal primers modified for 454 pyrosequencing (protocols.io dx.doi.org/10.17504/protocols.io.jgacjse), with unique molecular barcodes assigned to each reverse primer [55, 56]. Fungal-specific DNA was amplified (SSU 817f-1196r) using 0.25 μl of each primer (30 μM), 1.0 μL of BSA (10 mg mL-1), 3.0 μl of DNA template, and 22.5 μl Platinum PCR SuperMix (Invitrogen, Carlsbad, CA). Reactions ran for 30 cycles of 94°C for 45 seconds, 52°C for 30 seconds, and 72°C for 90 seconds with a hot start at 94°C for 10 minutes and final extension step at 72°C for 10 minutes. We pooled three PCR reactions for analysis, purification, and quantification; purified PCR products were then combined into an equimolar solution for downstream DNA pyrosequencing (protocols.io dx.doi.org/10.17504/protocols.io.jgacjse).

Our PCR products were sequenced by the Environmental Genomics Core at the University of South Carolina (Columbia, SC) on a Roche 454 Gene Sequencer (Roche 454 Life Sciences, Branford, CT) using titanium chemistry. We used a high-throughput pyrosequencing protocol and bioinformatics pipeline [54] for analyzing small-subunit rDNA of fungal communities. Sequenced amplicons were aligned and grouped into operational taxonomic units (OTUs) (i.e., fungal taxa) and taxa were defined as DNA sequences sharing ≥97% sequence identity; taxon level for this targeted portion of the small-subunit 18S rRNA gene has resolution at the fungal family level. Taxonomic information for each fungal taxon was determined using the BLAST algorithm [57] against identified sequences in Genbank and SILVA databases. For quality control purposes, we discarded any sequences < 400 bp and sequences with a phred quality score < 25. Samples were normalized to 562 sequences per sample, and any samples with fewer than 562 sequences or with unreadable barcodes were removed from our downstream analysis. Additionally, non-fungal sequences were manually removed following taxonomic assignment.

### Statistical analysis

#### Fragment size

To test our prediction that plant species richness declines with habitat fragment size, we performed a linear regression with fragment size as the independent variable and the number of plant species in a fragment as the dependent variable. We also evaluated whether fungal function declined with either fragment size or with the number of plant species in each fragment. In each of these cases, the independent variable was either fragment size or the number of plant species in a fragment, and the dependent variable was litter extracellular enzyme activity. Any significant decline in fungal function could be driven either directly by fragment size, or indirectly by plant species richness. Variables correlated with fragment size could have an effect on enzyme activity, thus we manipulated plant species richness to disentangle the effects of fragment size from those associated with the number of plant species in a fragment on fungi. Analyses associated with our plant litter manipulations enabled us to isolate the influence of plant diversity on fungal diversity and function.

#### Decomposition experiment

We conducted a linear regression to evaluate whether plant litter diversity controls fungal function in fragmented coastal sage scrub habitats. In this case, the independent variable was plant litter richness, and the dependent variable was percent litter mass remaining. A significant increase in the percent remaining of litter mass with decreasing litter richness would support our hypothesis.

In order to control for the effect of litter richness in the model and evaluate how percent litter mass remaining responded to litter richness, we conducted a generalized linear model with percent litter mass as the response variable, and litter richness and block as factors. We used a Shapiro-Wilk test for normality and assigned a Gaussian distribution with an identity link function, as responses were normally distributed. Additionally, we conducted a single sample t-test to compare the percent mass remaining from litter bags containing a single plant species with litter bags containing more plant species.

We conducted a linear mixed-model in order to address our second hypothesis that plant litter diversity controls fungal taxa richness. For examining fungal community shifts, we compared the relative abundance of Ascomycete fungal taxa across plant diversity levels in litter bags. In addition, we conducted another linear mixed-model to evaluate whether the percentage of litter mass remaining in our litter bags was related to fungal taxa richness; we assigned block as a random effect and nested plant litter richness within block to control for any effect of plant litter richness on fungal taxa richness and litter mass. A significant increase in percent litter mass remaining with decreasing fungal taxa richness would suggest that fungal taxa richness may be related to fungal functions like decomposition.

## Results

### Fragment size

**Fig 2:**
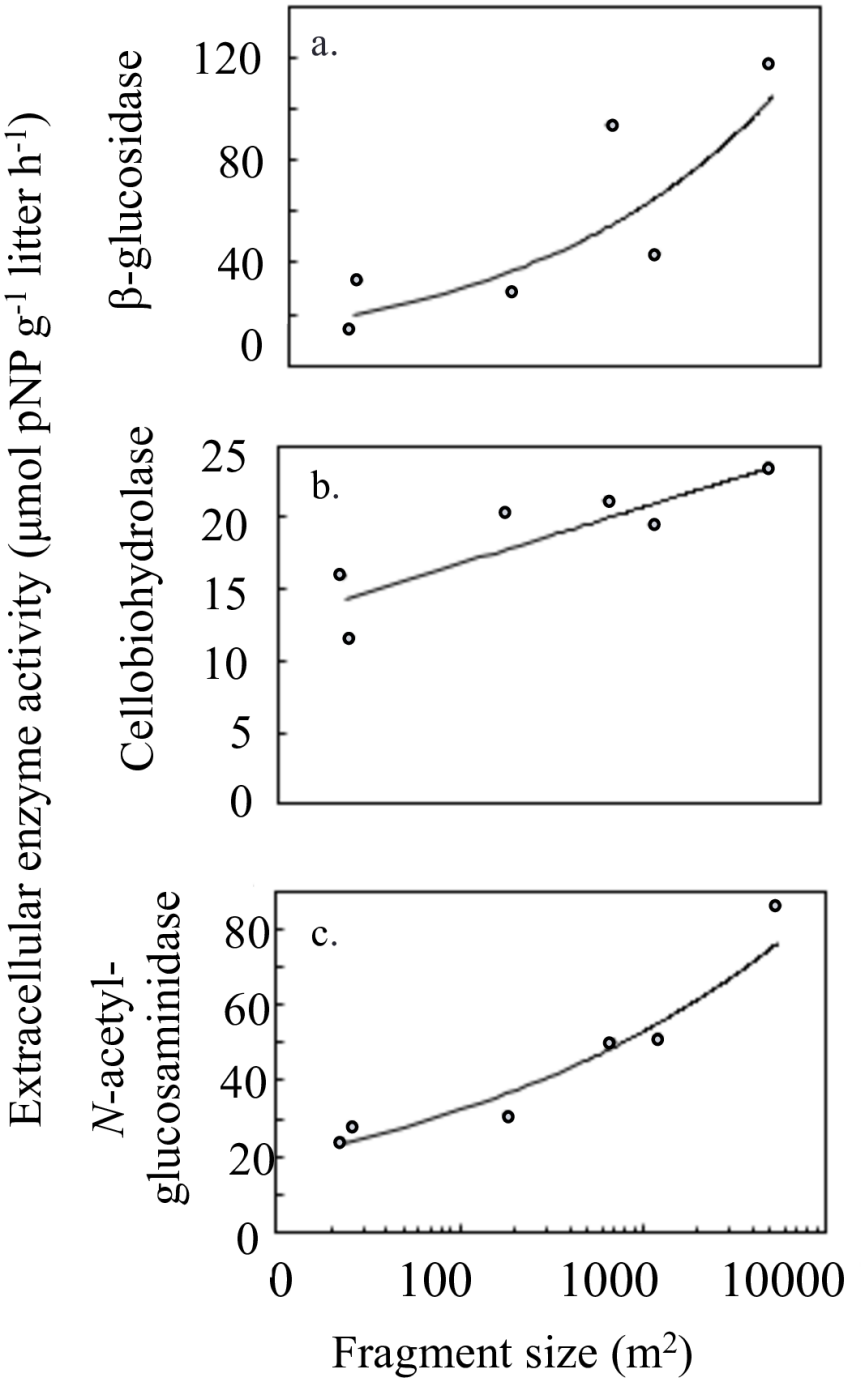
Extracellular enzyme activity and fragment size. Extracellular enzyme activity (μmol pNP g^-1^ litter h^-1^) in litter was significantly correlated with log fragment size (m^2^) for three extracellular enzymes involved in cellulose degradation, a. β-glucosidase (p = 0.035) and b. cellobiohydrolase (p = 0.022), and chitin degradation, c. *N*-acetyl-glucosaminidase (p = 0.002). Fitted line is shown with corresponding R^2^ value.

**Fig 3:**
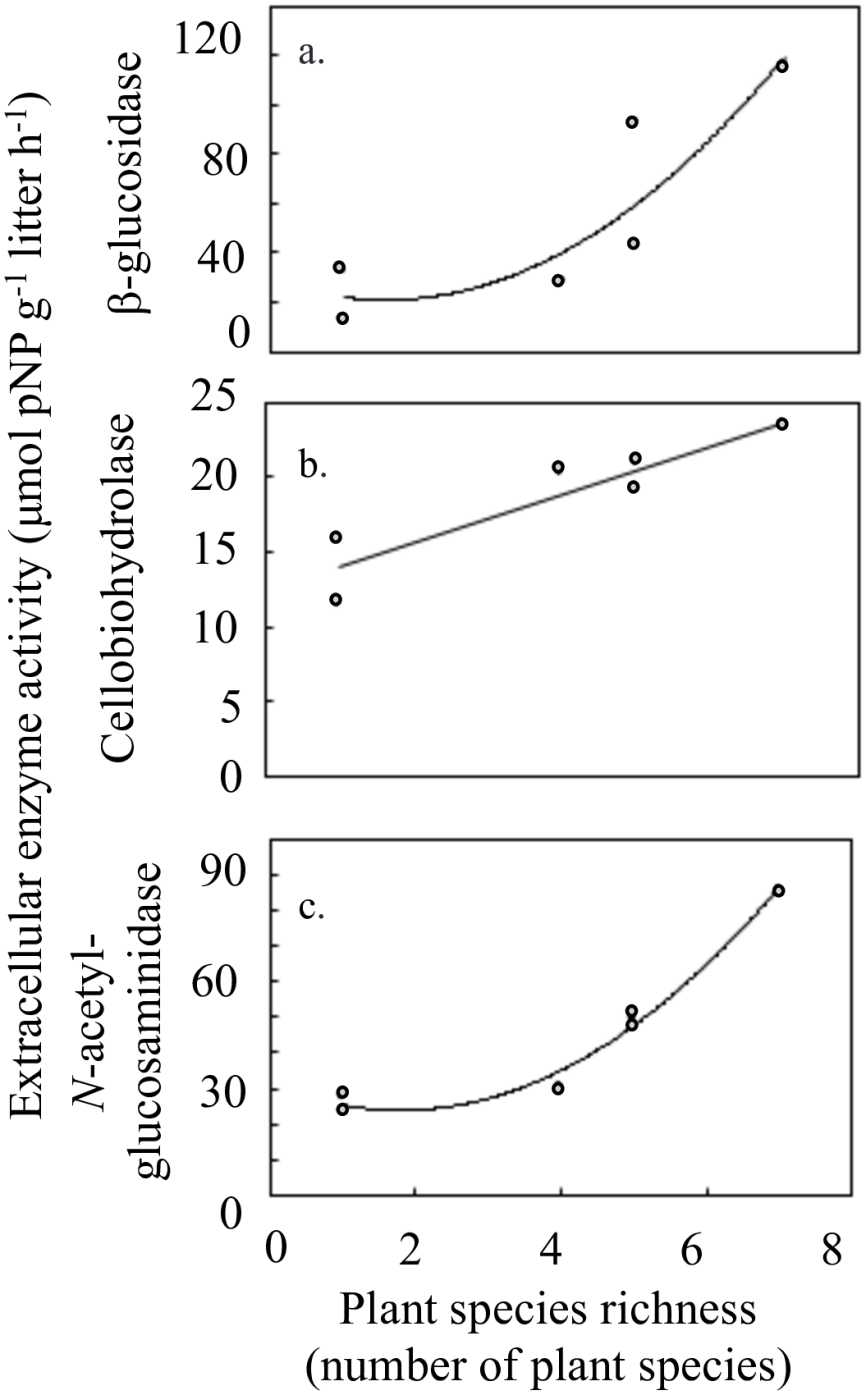
Extracellular enzyme activity and plant richness. Extracellular enzyme activity (μmol pNP g^-1^ litter h^-1^) in litter was significantly correlated with plant richness in the fragments for three extracellular enzymes involved in chitin and cellulose degradation: a. β-glucosidase (p = 0.017), b. cellobiohydrolase (p = 0.008), and c. *N*-acetyl-glucosaminidase (p < 0.001). Fitted line is shown with corresponding R^2^ value.

The species richness of plants in each fragment was correlated with fragment size (R^2^ = 0.976, p < 0.001). Furthermore, we observed that fungal function varied by fragment size and by plant species richness in the fragments. Specifically, litter extracellular enzyme activity was positively correlated with fragment size for three extracellular enzymes: β-glucosidase (R^2^ = 0.712, p = 0.035) (Fig 2a), cellobiohydrolase (R^2^ = 0.766, p = 0.022) (Fig 2b), and *N*-acetyl-glucosaminidase (R^2^ = 0.934, p = 0.002) (Fig 2c). Additionally, litter extracellular enzyme activity was positively correlated with plant species richness in the fragments for these same extracellular enzymes: β-glucosidase (R^2^ = 0.795, p = 0.017) (Fig 3a), cellobiohydrolase (R^2^ = 0.853, p = 0.008) (Fig 3b), and *N*-acetyl-glucosaminidase (R^2^ = 0.981, p < 0.001) (Fig 3c).

### Decomposition experiment

**Fig 4:**
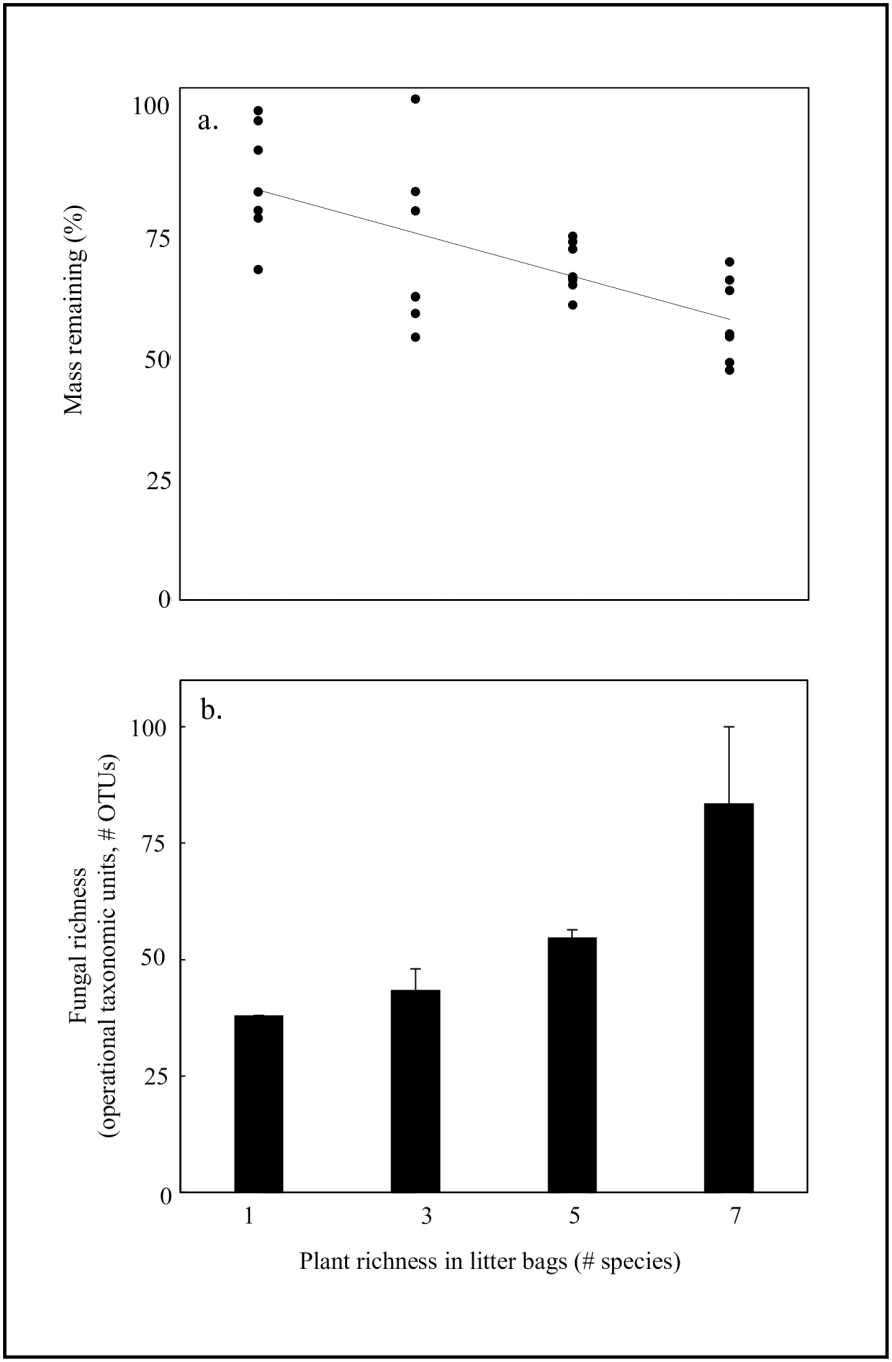
Percent mass remaining and fungal richness of field incubated litter bags. Percent mass remaining and fungal richness of litter bags containing different levels of plant litter richness after one year. Following field incubation, litter bags containing more species of plant litter had less plant litter mass remaining than litter bags with fewer plant species (R^2^ = 0.456, p = 0.0001, Fig 4a). Percent mass remaining values are shown for each sample in our litter diversity manipulation. Fungal richness increased with plant species richness in field incubated litter bags. Fungal richness (number of fungal taxa, i.e., operational taxonomic units or # OTUs) increased with greater numbers of plant species (# species) represented within litter bags (F_1,7_ = 13.64, p = 0.008, Fig 4b). Fungal taxa richness values (±1 SE) of mean fungal taxa richness by plant species richness within our plant litter diversity treatments.

**Fig 5:**
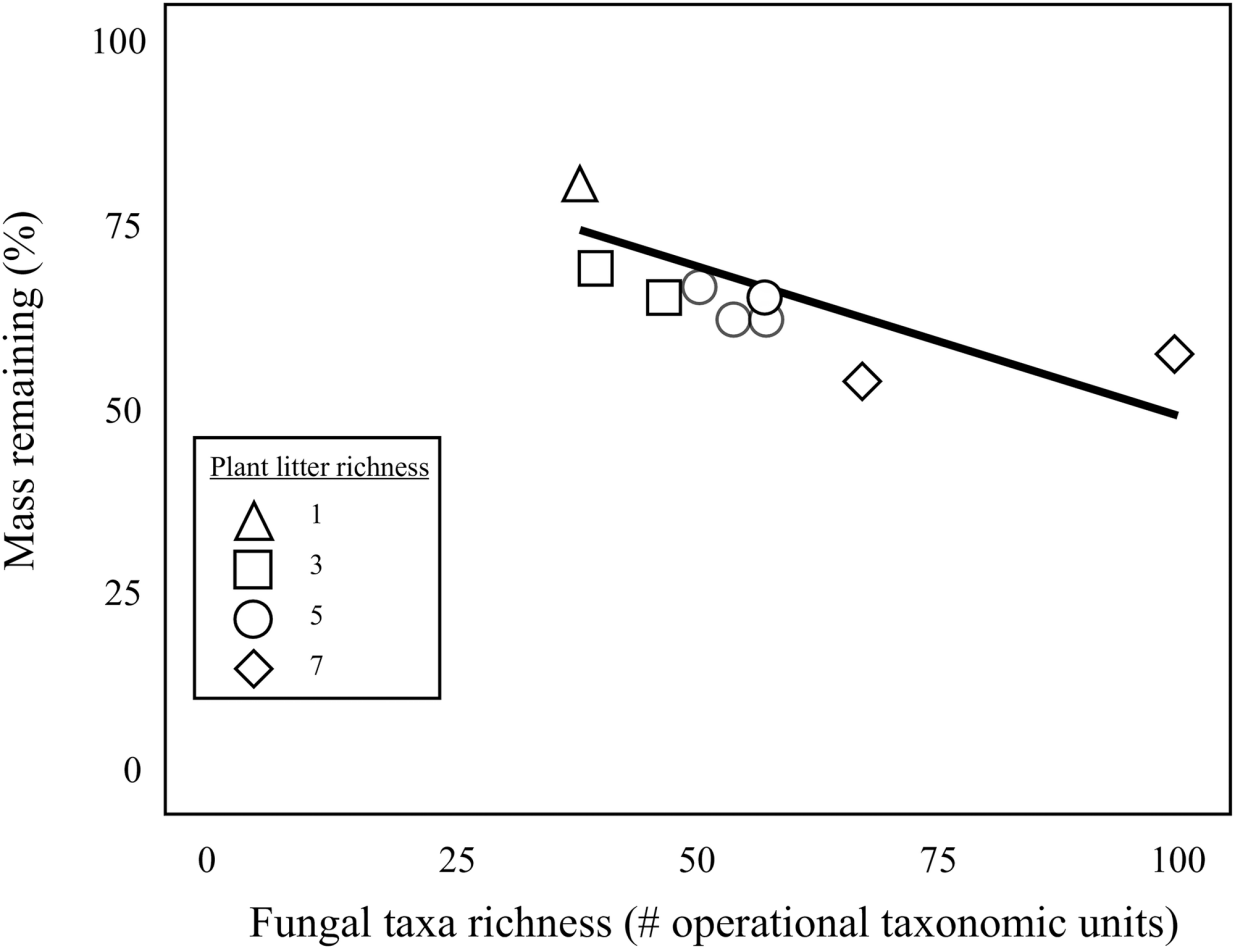
Percent mass remaining of field incubated litter bags was related to fungal taxa richness. Mass remaining (%) in litter bags was related to the number of fungal taxa (R^2^ = 0.516, p = 0.012) in these litter bags. Symbols represent levels of plant species richness of litter in litter bags.

After one year, litter bags containing more species of plant litter had less litter mass remaining than litter bags with fewer plant species (R^2^ = 0.456, F_1,26_ = 20.93, p = 0.0001, Fig 4a). The number of plant species contained in litter bags had an effect on percent litter mass remaining, such that single-species litter bags had significantly more litter mass remaining that litter mixtures (t = 24.83, p = 0.001). Results from our analyses suggest that sampling effects are not driving the differences we observed with litter mass loss. Litter bags with more plant species yielded significantly greater fungal taxa richness than litter bags with fewer plant species (F_1,7_ = 13.64, p = 0.008, Fig 4b). These findings supported our second hypothesis in that plant litter diversity controlled both fungal taxa richness and fungal function in our litter bags. A majority of fungal taxa identified from our litter bags were dikaryotic fungi, with 80% of fungal operational taxonomic units (OTUs) from Ascomycota and 13% of OTUs from Basidiomycota; the remaining 7% of OTUs were zoosporic taxa from either Chytridiomycota or Blastocladiomycota. Fungal community composition in litter bags shifted across litter richness levels. We detected a lower percentage of fungi from Ascomycota in litter-mixtures with more plant species than in litter bags containing fewer plant species (p = 0.011). Fungal richness was related to the percent litter mass remaining in litter bags. Litter bags with greater numbers of fungal taxa also had less litter mass remaining than those with fewer fungal taxa (R^2^ = 0.516, p = 0.012, Fig 5).

## Discussion

We examined the effects of habitat fragmentation on fungal function in a fragmented coastal sage scrub ecosystem and found reduced fungal function in smaller habitat fragments, suggesting that fragment size may indirectly influence fungal function via changes in plant diversity. Although our findings indicate that fungal function was related to both fragment size and plant diversity, other factors associated with fragment size may influence potential extracellular enzyme activity in litter from these fragments. While our study did not directly link decomposition and fungal diversity, when we isolated the effects that plant species richness had on fungal taxa richness and decomposition, we found that both fungal taxa richness and fungal function were controlled by plant litter diversity. Our findings highlight how fragmentation’s effect on coastal sage scrub plants may constrain both fungal processes and fungal communities within this ecosystem.

### Fragment size

Our finding that habitat fragment size was negatively related to plant species richness suggests that as intact coastal sage scrub ecosystems in southern California shrink, certain coastal sage scrub plants may compete for the limited habitat found within small fragments [58–60]. These reductions in plant litter diversity may reduce resource niche space for microbial decomposers [61, 62]. As litter inputs and nutrient availabilities change, this may in turn affect plant litter decomposition and fungal function in these ecosystems [63–66].

Overall, we found that fungal function significantly declined in more fragmented coastal sage scrub ecosystems, possibly because of reduced chitinase activity in litter from smaller fragments. *N*-acetyl-glucosaminidase mineralizes N from chitin [67], a polysaccharide present in fungal cell walls that is produced during fungal growth [68–71]. Thus, *N*-acetyl-glucosaminidase activity is often correlated with fungal biomass in plant litter [72, 73]. In our study, the observed decline in *N*-acetyl-glucosaminidase activity within litter from small fragments could indicate that either fungal growth, fungal turnover, or microbial function was diminished in coastal sage scrub fragments that contained fewer numbers of plant species.

We also observed that litter cellulase activity decreased with smaller coastal sage scrub fragments and with decreasing numbers of plant species, possibly because fragments with fewer plant species produced litter containing a limited variety of cellulose or other structural polysaccharides [74]. Low-diversity litter with fewer types of cellulosic compounds [75] may have diminished substrate availability for cellulolytic enzymes [76, 77]. Limited substrate availability may in turn inhibit the cellulytic potential of microbial communities by hindering cellulase gene expression [78]. Since these enzymes (β-glucosidase, cellobiohydrolase) are important for hydrolyzing compounds within cellulosic plant litter [79], this decrease in litter extracellular enzyme activity may thus have inhibited cellulose degradation in litter from small habitat fragments with less diverse plant communities.

Fragmentation’s constraints on plants and plant litter resources may indirectly affect decomposer fungal function because plant species differ in their chemistry and structure. Decomposing material from less diverse plant communities may contain a narrow range of resources [80], which could limit substrate availability and litter quality. The nutrient dynamics within less diverse or low-quality litter could potentially inhibit fungal function [80, 81]. Considering the high N cost and energy intensive process of enzyme synthesis, nutrient limitation may either constrain or promote extracellular enzyme activity; these resultant outcomes may depend on the particular enzymes or an availability of both complex substrates and assimilable resources [82–84]. Yet, our findings suggest that litter resources from small coastal sage scrub fragments may constrain interactions among microbial communities, extracellular enzymes, and substrates [85], lowering litter extracellular enzyme activity and disrupting microbial processes in plant litter substrates. If plant litter diversity constrains these interactions, small fragments characterized by less diverse resources may be less apt to support functionally diverse microbial communities.

### Decomposition experiment

We found that direct manipulations of plant litter diversity affected fungal taxa richness, implying that the effects of fragmentation on plant diversity may extend beyond the plant community to affect plant litter decomposers and detritivores [86, 87]. Thus, reductions in plant diversity following fragmentation, rather than fragmentation itself, may be one of the proximate causes driving changes in the fungal community by either reducing ecological niche space or by changing plant litter quality [88].

Diverse plant communities may provide more niche space for fungal taxa that exploit specialized ecological niches [89]. Fungi vary in their preferences for organic substrates, therefore changes in plant and fungal diversity could influence nutrient cycling in ecosystems [90]. In fact, highly diverse communities may host a range of species that respond differently to disturbance and thus help stabilize ecosystems [91, 92]. With more plant-fungal interactions and a greater variety of litter, diverse plant assemblages may provide sufficient resources for preventing losses of specialist or rare fungal taxa from fragmented ecosystems. Altogether, communities with greater plant diversity should produce more diverse litter resources and provide more ecological niche space, which may support greater numbers of fungal taxa.

Following our decomposition experiment, less litter mass remained within litter bags containing diverse plant litter, suggesting that these diverse fungal assemblages may have consumed more resources from mixed-litter bags than from single-species litter bags. This phenomenon may have occurred as a consequence of increased fungal diversity in these litter bags, as highly diverse communities are more likely to contain species that use resources efficiently because of sampling effects [93–95]. However, most plant species in our single-species litter bags decomposed less thoroughly than litter in our litter mixtures. This suggests that decomposition of plants in our litter mixtures may not have been driven solely by sampling effects, but potentially via *non-additive* effects, resulting from dynamics associated with diverse mixtures decaying more thoroughly over the same duration of time as a majority of these single-species alone [80].

In addition to fungal decomposer sampling effects, plant richness sampling effects [96] may result in diverse litter mixtures that contain highly productive plant species [97] or plant species that rapidly decompose [98, 99]. In other ecosystems, plant material in less diverse litter mixtures may contain more labile substrates, which could potentially require fewer fungal taxa to achieve similar decomposition rates. However, because more diverse litter mixtures in our study also contained greater numbers of fungal taxa, synergy between plant litter richness and fungal taxa richness may have enhanced organic substrate turnover. As fungal taxa specialize on particular organic substrates, this increased fungal diversity may have consequently accelerated decomposition rates [90].

Our study provides evidence that both habitat fragment size and the number of plant species in a fragment affect fungal function, yet it has some limitations. For instance, our study was conducted within only one ecosystem, and thus our interpretations are limited to coastal sage scrub ecosystems. Additionally, while we opted to focus our study on fungi, other microbes like bacteria contribute to soil processes in fragmented habitats [11, 12]. Beyond fungi, investigating other microbial responses to fragmentation was outside the scope of this study. Nevertheless, because fungi play a key role in biogeochemical cycles [15, 100], determining their functional response is especially important for predicting how coastal sage scrub ecosystems respond to fragmentation. It is worth noting that abiotic factors related to soil moisture and landscape topography, such as slope or aspect, or characteristics related to plant biomass or richness, such as the percent vegetative cover or the lability of plant litter components, may independently influence fungal function. However, results from our plant litter manipulation suggest that fungal taxa richness may play a role in nutrient cycling in remnant coastal sage scrub habitats.

## Conclusion

Our results suggest that reduced plant diversity may constrain both fungal taxa richness and fungal function in fragmented coastal sage scrub ecosystems. We found that plant diversity in coastal sage scrub ecosystems was particularly susceptible to fragmentation, which may ultimately limit fungal metabolic activities. Larger fragments containing multiple litter types may provide sufficient microhabitat heterogeneity for supporting greater numbers of functionally diverse fungal taxa. These diverse fungal communities may efficiently exploit litter resources, which may increase decomposition. Therefore, as fragmentation directly reduces plant diversity it may also indirectly influence fungal function. Altogether, our findings provide evidence that, like many macroscopic taxa, fungi may also be affected by human-driven habitat fragmentation via direct effects of fragmentation on plants. These data have crucial implications for management of coastal sage scrub ecosystems. For instance, restoration methods that aim to restore diverse vegetation communities within larger fragments may be especially effective at improving the functional capacity of degraded sites. This study underscores the importance of both reducing habitat fragmentation and maintaining diversity when restoring ecosystems.
